# Follow-Up of Head and Neck Cancer Survivors: Tipping the Balance of Intensity

**DOI:** 10.3389/fonc.2020.00688

**Published:** 2020-05-06

**Authors:** Petr Szturz, Carl Van Laer, Christian Simon, Dirk Van Gestel, Jean Bourhis, Jan B. Vermorken

**Affiliations:** ^1^Medical Oncology, Department of Oncology, Lausanne University Hospital (CHUV), Lausanne, Switzerland; ^2^Department of Otolaryngology and Head and Neck Surgery, Antwerp University Hospital, Edegem, Belgium; ^3^Faculty of Medicine and Health Sciences, University of Antwerp, Antwerp, Belgium; ^4^Department of Otolaryngology - Head and Neck Surgery, Lausanne University Hospital (CHUV), Lausanne, Switzerland; ^5^Department of Radiotherapy, Institut Jules Bordet, Université Libre de Bruxelles, Brussels, Belgium; ^6^Radiation Oncology, Department of Oncology, Lausanne University Hospital (CHUV), Lausanne, Switzerland; ^7^Department of Medical Oncology, Antwerp University Hospital, Edegem, Belgium

**Keywords:** head and neck cancer, survivorship, surveillance, recurrence, metastasis, second primary tumor, late toxicity, quality of life

## Abstract

The traditional concept of post-treatment surveillance in head and neck cancer patients relies on examinations directed at early detection of disease recurrence and/or second primary tumors. They are usually provided by ear, nose and throat specialists with complementary input from radiation oncologists and medical oncologists. Emerging evidence underscores the importance of monitoring and effective management of late adverse events. One of the major drawbacks is a lack of prospective controlled data. As a result, local institutional policies differ, and practice recommendations are subject to continuing debate. Due to the economic burden and impact on emotional comfort of patients, intensity and content of follow-up visits are a particularly conflicting topic. According to the current evidence-based medicine, follow-up of head and neck cancer patients does not prolong survival but can improve quality of life. Therefore, an approach giving priority to a multidisciplinary care involving a speech and swallowing expert, dietician, dentist, and psychologist may indeed be more relevant. Moreover, on a case-by-case basis, some patients need more frequent consultations supplemented by imaging modalities. Human papillomavirus positive oropharyngeal cancer tends to develop late failures at distant sites, and asymptomatic oligometastatic disease, especially in the lungs, can be successfully salvaged by local ablation, either surgically or by radiation. The deep structures of the skull base related to the nasopharynx are inaccessible to routine clinical examination, advocating periodic imaging supplemented by nasofibroscopy as indicated. Anamnesis of heavy smoking justifies annual low-dose computed tomography screening of the thorax and intensive smoking cessation counseling. Finally, some cancer survivors feel more comfortable with regular imaging, and their voice should be taken into consideration. Future development of surveillance strategies will depend on several variables including identification of reliable predictive factors to select those who could derive the most benefit from follow-up visits, the availability of long-term follow-up data, the results of the first randomized trials, resource allocation patterns, infrastructure density, and the therapeutic landscape of locally advanced and recurrent and/or metastatic disease, which is rapidly changing with the advent of immune checkpoint inhibitors and better utilization of local approaches.

## Introduction

Aiming at different aspects of post-treatment monitoring, follow-up has always been an integral part of modern oncology care. In head and neck cancer, the target group of patients consists of those who underwent curative therapy for early or locoregionally advanced disease, although this paradigm may soon be changing. Distant metastases have traditionally portended a dismal prognosis with median overall survival of <1 year. The recent advent of immunotherapy together with better integration of local ablative modalities holds the promise of an improved, yet still rare long-term disease- and treatment-free survivorship even in patients initially managed with palliative intent (1, 2). The concept of post-treatment surveillance is based on the following two premises. First, compared with self-referral, it allows early detection of an abnormality. Second, early detection, compared with late diagnosis, preferably in the asymptomatic stage, leads to improved outcomes. However, neither of these hypotheses has been supported by strong evidence, partly due to ethical reasons related to the design of the control arm which should be ideally based on very reduced or no-follow-up approaches. Until present, no randomized trial has successfully compared two different follow-up strategies or a given follow-up protocol with no surveillance. Nevertheless, most of the internationally recognized societies recommend an intensive search for a locoregional failure during the first 2–3 years corresponding with the biologic behavior of recurrent disease. As a result, appointments can be as frequent as every 1–3 months at the beginning, progressively dropping off on each consecutive year, so that patients are usually seen annually after 5 years (3). Of note, these guidelines are not uniformly accepted but rather adapted by practicing physicians sometimes to their personal beliefs such as the notion about the allegedly beneficial use of routine positron emission tomography/computed tomography scan imaging (PET/CT) in asymptomatic cancer survivors (4).

The key question to address is whether available data sufficiently endorse intensive follow-up protocols or whether we can decrease the frequency of appointments without harming our patients. In this respect, disease-oriented examinations focusing on tumor detection should be distinguished from patient-oriented appraisal of late adverse events. In this paper, the term “intensive follow-up” partly overlaps with the general impression of current follow-up protocols, but due to existing variations among different centers, it should rather be perceived as a relative concept allowing us to discuss different comparisons with “less intensive” approaches. Another intriguing issue is possible personalization of surveillance based on disease subsite, biological characteristics, and molecular biomarkers or patient risk factors. The disease group of interest here comprises primarily squamous cell carcinoma of the head and neck (SCCHN), i.e., of the oral cavity, oropharynx, larynx, and hypopharynx, and nasopharyngeal cancer, but the obtained findings can be, to a certain extent, extrapolated to some less frequent entities of the head and neck region due to a paucity of data relevant to rare diseases. The presented conclusions do not apply to primary response assessment in SCCHN by means of clinical evaluation and imaging within 3 months after radiotherapy or chemoradiotherapy, which belong to the standard of care and have been covered elsewhere (5).

## Reporting and Interpretation of Follow-Up Studies

When evaluating different surveillance programs, the key objective is to determine how many patients could benefit from early detection of recurrence and/or second primary tumor. Overall survival remains the best indicator of that. Secondary endpoints include proportion of detected recurrences or second primary tumors (pick-up rate), proportion of patients eligible for a curative approach and of those who finally undergo such treatment, quality of life, and early detection rate of late adverse events and comorbid conditions. As opposed to clinical trials exploring a new therapeutic modality, the hallmark of surveillance studies are the characteristics of follow-up visits which may influence the actual intervention. Therefore, a recurrence rate *per se* reported in clinical studies does not sufficiently describe the effectiveness of surveillance programs analyzing the utility of different follow-up schedules and of the respective modalities used (physical examination, endoscopy, imaging, blood tests, etc.).

A rigorous interpretation of the results starts with collecting the pick-up rate data and distinguishing between symptomatic and asymptomatic cases followed by identifying the proportion of eligible and intervened patients, the latter of which qualify for comparative survival assessments. Important is to avoid confusion with self-referral which informs us about symptomatic patients examined at off-schedule visits and corresponds thus with a no-follow-up approach. Typical symptoms necessitating further evaluation include new onset or worsening of pain, hoarseness, and a lump in the neck. Analogously to screening programs, the calculated benefit of a given follow-up protocol vs. self-referral can be overestimated by lead-time and length-time biases. In addition, two further aspects should be addressed. Cost-effectiveness calculations usually focus on the amount of costs necessary to detect one recurrence. The obvious limitation is the lack of information on the real benefit reflected by the resulting impact on overall survival. The second point is quality of life characterized by several contributing factors, not only by disease recurrence, but also by second primary tumors, late adverse events, and lifestyle behaviors (mainly smoking and alcohol intake).

Next to elaboration of the optimal timing and procedures of the follow-up routine, further efforts urge to define patient subgroups who benefit most. In this respect, life expectancy, disease stage, primary site (oral cavity, larynx, and the subdivisions of pharynx), and molecular markers such as human papillomavirus (HPV) status or p16 status as its surrogate marker belong to commonly used criteria in clinical practice. Of note, more intensified surveillance is often prescribed to patients initially presenting with advanced disease. While in these cases, recurrences are indeed more frequent than in early stage head and neck cancer, they are less likely to be successfully salvaged (6, 7).

## Arguments Against Intensive Follow-Up

There has been weak evidence of improved outcome resulting from a salvage intervention of recurrences detected at routine follow-up visits when compared with those detected at self-referral. In one retrospective study, 428 patients with SCCHN were treated between 1979 and 1983 and followed for 84–126 months. The follow-up schedule consisted of a locoregional examination and medical history performed regularly at given time points with a decreasing intensity for a total of 10 years (6x during the first year, then 4x and 3x during the second and third years, respectively, then 2x until the end of the fifth year and annually afterwards). An annual chest X-ray was mandatory. The authors found a significantly better mean survival (58 vs. 32 months, *p* < 0.05) after detection of an event (i.e., recurrence or second primary tumor) with a routine follow-up (185 events in 6,350 appointments, pick-up rate 1 in 34) vs. self-referral (20 events in 54 appointments, pick-up rate 1 in 2.7), respectively. The corresponding cure rates were 1 in 78 and 1 in 6.8 appointments, respectively. Of note, 67% of events detected at routine follow-up were symptomatic. The study, thus, excels in providing us with very detailed and rigorous reporting, but due to the small number of patients in the self-referral cohort, the results should be regarded with caution (8). In addition, quantitatively more data, albeit still retrospective, did neither confirm such survival benefit nor find a correlation between follow-up intensity and survival (7, 9–11). The relevance of intensive follow-up is further undercut by the fact that the majority of recurrences (56–85%) are symptomatic and therefore potentially amenable to a successful self-referral (7, 8, 10–16). The real-world setting brings another important factor to the forefront, i.e., compliance. According to different author groups, non-adherence to surveillance protocols varies, being more often found in patients with small primary tumors, who live far from a hospital and continue to smoke (6). Nevertheless, this does not seem to have influence on survival outcomes (7).

It can be argued that the reason why the majority of recurrences are symptomatic is the insufficient detection capacity of a physical examination supplemented by endoscopy as indicated. At first glance, this seems to be a credible statement since very small neoplastic changes remain clinically silent. In this respect, biochemical tumor markers are commonly prescribed in oncology practices with varying degree of supporting evidence. In SCCHN, this diagnostic approach lacks sufficient sensitivity (17). The only exception could be Epstein-Barr virus deoxyribonucleic acid (DNA) analysis in nasopharyngeal cancer survivors with emerging new data on HPV cell-free DNA monitoring in viral-related oropharyngeal cancer (18, 19). As none of these has been standardized for routine clinical use yet, much attention has been paid to imaging modalities. Formerly recommended annual chest x-rays capture only a minority of lung tumors in their asymptomatic growth phase. According to a recent meta-analysis, a chest X-ray misses about 25% of cancer lesions (20). Notwithstanding the diagnostic pitfall when differentiating a SCCHN lung metastasis from a lung primary, most of the cases diagnosed by plain radiography correspond to head and neck cancer dissemination (21). Computed tomography alone or in combination with PET imaging indeed improves detection of asymptomatic lesions. Surveillance imaging by means of PET/CT has very good sensitivity and negative predictive value but only moderate specificity and positive predictive value (22). On the contrary, conventional evaluation by a physical examination, chest X-ray, CT, and magnetic resonance imaging has lower sensitivity but higher specificity (23). Positron emission tomography was shown to influence treatment decision in about 1 out of 3 cases. Unfortunately, no impact on survival has been demonstrated yet, probably due to the low yield of hypermetabolic lesions (about one third at maximum), of which not all are amenable to surgery and not all of the amenable cases finally undergo a resection (22, 24, 25). Illustrative to that is a recent retrospective study of 326 patients in which a clinical and radiological follow-up involving periodic CT, magnetic resonance imaging, and PET scan identified more recurrences in the asymptomatic phase than were patient-detected cases, which were symptomatic at a scheduled appointment or revealed during an unplanned, symptom-driven consultation. However, the proportion of patients eligible to a curative treatment remained comparable as well as their survival outcomes (26).

The choice of therapeutic approach depends on clinical setting. In oligometastatic disease (1–5 metastases), quantitatively more data support surgery which remains thus the gold standard in this scenario (5-year overall survival about 30%) (2, 27). As a viable alternative to an invasive procedure, stereotactic radiotherapy yields similar outcomes, although we lack a direct comparison between the two modalities (28). In the rare cases of solitary metastases, both surgery and radiotherapy show the maximum efficacy with a 5-year survival rate of up to 56% (29). A different situation exists when locoregional recurrence develops because the respective anatomical region was already subjected to prior interventions. The ensuing consideration are survival rates in those who undergo a salvage procedure by different modalities. Surgical resection of locally and/or regionally recurrent disease, if technically possible, yields the best results with a 5-year overall survival of up to 39%, particularly in early disease and laryngeal primary (30, 31). Interestingly, such outcomes seem to be preserved even after operating on a second recurrence (32) On the other hand, definitive re-irradiation in patients with an unresectable disease, with or without chemotherapy, should be delivered with caution. The low survival rates of 10–30% at 2 years are further dampened by 40% of severe late toxicities and 10% treatment-related mortality (33). More recently, comprising nine centers from the United States, the Multi-Institution ReIrradiation (MIRI) Consortium analyzed about 500 patients with a resectable or unresectable recurrence or second primary tumor treated by radiotherapy or more commonly by chemoradiotherapy. At 2 years, overall survival reached up to 35% with severe acute toxicity not exceeding 22%. Based on a recursive partitioning analysis (RPA) of time from first course of radiation, resectability, and organ dysfunction, 3 prognostic subgroups were defined. Of note, RPA class III patients (i.e., time from first-course radiotherapy of 2 years or less and the presence of organ dysfunction) are not ideal candidates for protracted chemoradiation regardless of resection status (34–36).

Unfortunately, the majority of patients with recurrent and/or metastatic SCCHN are eligible neither for surgery nor radiotherapy, and the remaining options are limited, questioning thus the role of intensive follow-up. Even the most potent systemic treatment combining a chemotherapy doublet with an immune checkpoint inhibitor should be regarded as a palliative measure. The expected median overall survival only slightly exceeds over 1 year, albeit with a chance of long-term survivorship for a minority of patients (perhaps 10–20%) (37). Noteworthy, early initiation of systemic therapy to improve outcome has not been based on any strong evidence, and recommended factors guiding our decision include disease kinetics, risk of treatment-related toxicities, and presenting symptoms (38).

Last but not the least, follow-up visits contribute to healthcare resource consumption. Although we lack direct comparisons between different surveillance programs, unjustified follow-up visits are not cost-effective. According to a 1998 publication, the estimated costs per detected recurrence or second primary tumor ranged from $2,587 for non-intensive to $49,242 for intensive follow-up (39). The proponents of intensive follow-up might argue that the results of such analyses should be put into the context of modern immunotherapy currently approved for palliative treatment of recurrent and/or metastatic SCCCHN. As an example, it was shown that about $300,000 may be needed to invest to gain one quality-adjusted life-year (QALY) when treating with second-line nivolumab relative to a standard-of-care chemotherapy or cetuximab (40).

## Arguments in Support of Intensive Follow-Up

Besides recurrent disease, follow-up visits address the risk of second primary tumors sharing the same risk factors as most of the head and neck cancer cases, i.e., tobacco and alcohol. Second primary tumors occur at an average rate of 2–4% per year with a cumulative incidence of 5–35% predominantly in the head and neck region if the index cancer was localized in the oral cavity and oropharynx, but also at other sites as the lungs in patients with a past medical history of laryngeal or hypopharyngeal cancers and in the esophagus. Generally, they have better prognosis than recurrent tumors (41, 42). Hypopharyngeal cancer is associated with the highest probability of second primary tumors (6, 42). The risk of metachronous tumors should be a sufficient reason for smoking cessation and has an important implication for periodic CT scans in heavy smokers. According to the National Lung Screening Trial, there is level I evidence for reduced lung-cancer mortality in persons between 55 and 74 years of age who stopped smoking 15 years ago or earlier and who have a strong history of tobacco smoking of at least 30 pack-years when subjected to annual low-dose CT screening (43). Moreover, cancer survivors are advised to participate in colorectal, breast, and cervical screening programs. Less evidence is available for screening interventions to prevent other malignancies, and attentive symptom-directed investigations should be pursued in these cases.

The role of imaging methods differs according to the site of primary tumor, which impacts on screening of both locoregional and distant recurrences, and we will discuss these two clinical scenarios separately. Due to the inaccessibility of the deep structures of the skull base to routine clinical examination, periodic imaging, supplemented by nasofibroscopy if need be, is warranted in nasopharynx cancer survivors. Similarly, post-radiotherapy changes in laryngeal tissues may necessitate supplementary endoscopy or imaging in these patients (44). Frequent early endoscopic examinations are also suggested in patients who underwent endoscopic surgery, either transoral laser microsurgery (TOLS) or transoral robotic surgery (TORS), because more extensive resection is often feasible in local recurrences (author experience). In the majority of SCCHN cases, distant metastases are not the predominant type of failure except for the following two subsites, nasopharyngeal cancer and human papillomavirus positive oropharyngeal cancer. Here, additional efforts have been exerted to define appropriate surveillance.

Human papillomavirus positive oropharyngeal cancer represents a distinct entity characterized, among other things, by younger age of patients, usually a long-term survivorship, and a specific recurrence pattern. As opposed to its HPV-negative counterparts and other SCCHN cases, hematogenous dissemination is the prevailing type of failure and occurs over a longer period of time. While in HPV-negative disease, the majority of distant recurrences develop within the first 2 years, more than 10% of HPV-positive oropharyngeal cancer cases, notwithstanding an overall better distant control of around 85–90%, continue to metastasize beyond 3 years and a smaller proportion even after 6 years from diagnosis. Overall survival after distant failure is longer in HPV-positive patients, where oligometastatic disease of the lungs, i.e., one to five lesions, portends a potential for curative management in about one third of patients, primarily using surgery or radiotherapy (45–47). Importantly, most of the distant recurrences detected by surveillance imaging, such as PET/CT, are asymptomatic (48). Taken together, these findings support the notion that HPV-positive oropharynx cancer patients can also benefit from intensive follow-up involving imaging methods. Another head and neck cancer subsite known for the prevailing pattern of distant failure is the nasopharynx with analogous consequences in terms of radiological surveillance, as in the case of HPV-positive oropharyngeal cancer, in addition to the recommended periodic imaging to detect local recurrences as alluded to above (49, 50). Here, patients with pulmonary metastases alone may experience longer survival if local ablation is combined with systemic treatment (51).

As alluded to above, an essential part of post-treatment surveillance, especially in those treated with a bimodality or trimodality approach, consists of an active search for and management of late side effects which may sometimes have equally debilitating consequences as re-appearance of malignant outgrowth (52, 53). Among the most common complications, resulting from the treatment but also from the initial disease spread, are problems with swallowing, sometimes accompanied by pain, weight loss, xerostomia, and dental issues. Further impact on the quality of life may have unrecognized or untreated hypothyroidism, depression, carotid stenosis, and problems with speech and hearing. A secondary analysis of three chemoradiotherapy trials revealed a crude rate of late toxicity of 43%, mostly in terms of pharyngeal and laryngeal toxicity. Predisposing factors were identified on multivariate analysis including older age, advanced T stage, primary site in the larynx or hypopharynx, and neck dissection after completion of chemoradiotherapy (53).

More recently, in a meta-analysis of aggregate data from 31 prospective trials exploring the standard concurrent chemoradiotherapy with three-weekly high-dose cisplatin, overall prevalence of severe late toxicity was about 20% with xerostomia, dysphagia, and subcutaneous fibrosis each not surpassing 10%. Pooled rates of grade 1–2 xerostomia after definitive and postoperative chemoradiation were 59 and 81%, respectively (54). However, it should be kept in mind that reporting of late adverse events often suffers from inconsistency and incompleteness. As an example, possibly reflecting an increase in delayed adverse events, the updated results of the Radiation Therapy Oncology Group (RTOG) 91-11 trial suggested worse long-term outcome in the standard chemoradiotherapy arm as compared to the group treated with induction chemotherapy followed by radiotherapy alone (55). Looking retrospectively at long-term side effects in 10-year survivors, another author group identified about 20% of patients, treated with conventional (2-dimensional) radiotherapy with or without chemotherapy, requiring permanent gastrostomy tube placement at a median of 5.6 years (range 0–20.3) and about the same proportion of cases developing osteoradionecrosis at a median of 7.2 years (range: 0.5–15.3) (56). Fortunately, modern radiotherapy techniques, such as Intensity-Modulated Radiation Therapy (IMRT), are expected to reduce these unfavorable late toxicity rates (57, 58).

Delayed side effects have a substantial influence on quality of life and a properly conducted follow-up should involve speech and swallowing evaluation for timely interventions. Appearing with a variable time of onset, hypothyroidism, either subclinical or as clinically overt disease, is a frequent side effect of radiotherapy, necessitating thyroid-stimulating hormone testing at least once per year (59). Head and neck cancer survivors fear recurrence and need emotional support. Contrasting with underutilization of mental health services, depression is relatively common in this population with a prevalence of about 15%. Factors associated with post-(chemo)radiotherapy depressions encompass tracheostomy or gastrostomy tube and continued smoking (60). Psychological distress occurs after primary surgery at comparable rates (61, 62). Together with anxiety and fatigue, depression has one of the strongest correlations with quality of life (63). Of note, head and neck cancer survivors have the second highest mortality rate from suicide which is twice as high as compared with other oncology diagnoses and more than 3 times higher than the general US population (64, 65). In this context, the importance of social support and its periodic evaluation should be underlined.

Finally, in the era of shared medical decision making, the patient's voice should also be heard. Albeit still scarce and to a certain extent contradictory, the available retrospective data do not equivocally endorse that patients demand a less intensive follow-up protocol. On the contrary, they seem to feel more comfortable with regular imaging (66–69). However, the feeling of reassurance and satisfaction with the care they get may in some cases be counterbalanced by harmful aspects of such close surveillance including scan-associated distress, ultimately leading to a worse quality of life, excessive radiation exposure, unnecessary additional work-up, low cost-effectiveness, and even distraction from other recommended follow-up procedures (22, 70).

## Finding a Compromise

In the absence of randomized prospective evidence, our decision making depends on retrospective data analyses and expert opinion. On the one hand, the economic and resource burden imposed by unnecessary follow-up visits on the health-care system is considerable, on the other hand, the multifaceted and complex character of head and neck cancer advocates frequent consultations to address the diverse issues these patients face. A possible solution could be to replace some of the routine physical examinations by a specific appraisal of nutritional, swallowing, dental, and psychosocial status. Especially good-prognosis young patients, such as those with HPV-related oropharyngeal carcinoma or nasopharyngeal carcinoma, could benefit from such approach, along with an adequate imaging surveillance. According to this conception, follow-up should not be diminished but rather reorganized and rationalized to a more cost-effective model which does not primarily limit the costs but increases efficacy by improving quality of life, offering a better rehabilitation, and enhancing return to work. In this respect, new cost-effective options such as nurse-led follow-up care may even be beneficial in terms of health-related quality of life (71). Due to the respective competences and accreditation for clinical examination, the head and neck surgical discipline has a major role in the surveillance of patients who have been treated for head and neck cancer. However, a holistic approach to patient follow-up should be pursued whenever possible. It can be offered by a dedicated team consisting not only of an ENT specialist, but also a medical and radiation oncologist, a specialized nurse, a swallowing expert, a dietician, a dentist, and a psychologist.

## Future Outlooks

**Figure 1:**
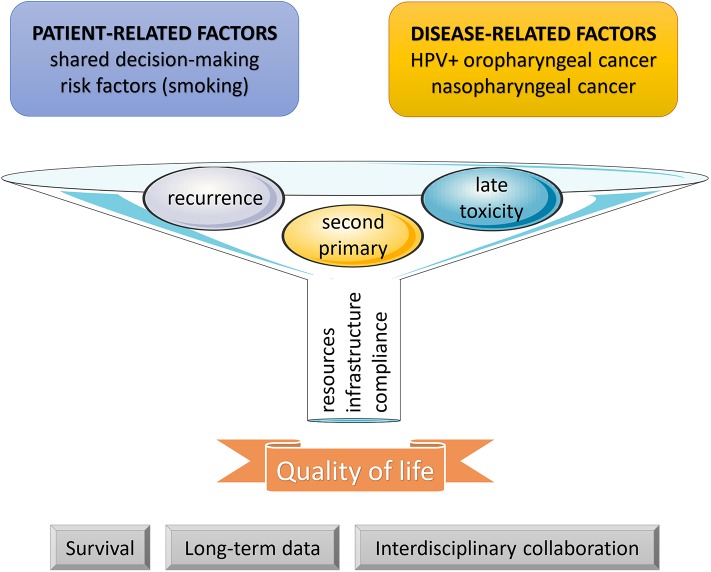
Follow-up funnel. During the post-treatment phase, surveillance is indicated in all cancer patients. As defined by patient- and disease-related factors, a more intensive approach may be considered. In head and neck cancer survivors, the three main goals of surveillance include an early detection and management of recurrences, second primary tumors, and late adverse events. The results depend on patient compliance and available resources and infrastructure. Until present, improvement in quality of life has remained the strongest outcome, and further efforts are needed to clarify the impact on survival, to collect sufficient evidence from long-term data, and to structuralize interdisciplinary collaboration between all professional stakeholders.

At this moment, improved quality of life depending on early detection of late toxicities and their appropriate management remains the strongest advantage of surveillance in head and neck cancer patients after treatment termination, albeit not supported by prospectively controlled evidence (Figure 1). Data on the outcomes of recurrence management are still scarce and do not allow us to make firm conclusions. Informative in this respect might be the currently ongoing SURVEILL'ORL (NCT03519048) and HETeCo (NCT02262221) trials aiming to randomly assign a total of almost 1500 participants between conventional surveillance and follow-up strategies intensified mainly by imaging methods after curative therapy of head and neck cancer with a primary outcome measure of overall survival (SURVEILL'ORL) and cost-effectiveness (HETeCo). Besides that, two promising techniques have recently emerged that will probably contribute to shaping oncology care in the future. The first are Electronic Patient Reported Outcomes (ePROs), allowing real-time symptom monitoring and even offering a survival benefit as demonstrated in a lung cancer study, in which after introduction ePROs, median overall survival rose from 13.5 to 22.5 months (72). A second innovative approach consists of circulating tumor cells enumeration which has been found associated with an increased risk of distant metastases, thus harboring potential for their early detection during follow-up (73). In theory, the latter technique may also open new avenues for experimental preemptive treatments.

Another crucial aspect impacting on future evolution of surveillance protocols involves the changing landscape of treatment-related morbidity. Together with advances in surgery (robot-assisted interventions) and radiotherapy (IMRT, stereotactic procedures, and proton therapy), the advent of the new class of immunotherapeutic agents (immune checkpoint inhibitors such as pembrolizumab and nivolumab) has been exerting powerful influences on the therapeutic landscape of head and neck cancer. It will not take long before we start following patients who were treated with these medicines in the past, most commonly in the context of locoregionally advanced disease. In addition, long-term survivors of recurrent and/or metastatic disease, who are not on immunotherapy any more, represent an emerging group of patients requiring a more focused care. Given the immune-related adverse events which are still difficult to predict and can even be delayed appearing after the treatment has already been terminated, an additional work-up during surveillance might be warranted. Consequently, the concept of follow-up will need to be rethought, tipping the balance of intensity once again.

## Author Contributions

All authors participated in the preparation of this manuscript.

## Conflict of Interest

PS: Advisory relationships: Merck-Serono, Servier, BMS. Honoraria received: Merck-Serono. CS: consulting and advisory services, speaking or writing engagements, public presentations: Pfizer, Merck. Direct research support: Roche. Non-financial interests: PI of EORTC 1420, non-remunerated member of the EORTC HNCG. DV: Advisory board/Honoraria received: Sanofi, Accuray, Merck, Pfizer, and Novartis. JB: Advisory board: MSD, BMS, Merck, Astra-Zeneca. JV: Has had in the last 3 years or has consulting/advisory relationships with: Immunomedics, Innate Pharma, Merck-Serono, Merck Sharp & Dome Corp, PCI Biotech, Synthon Biopharmaceuticals, Debiopharm, Cue Biopharma, and WntResearch and has received lecture fees from Merck-Serono, MSD, and BMS. The remaining author declares that the research was conducted in the absence of any commercial or financial relationships that could be construed as a potential conflict of interest.
